# Self-supervised learning framework for efficient classification of endoscopic images using pretext tasks

**DOI:** 10.1371/journal.pone.0322028

**Published:** 2025-05-08

**Authors:** Shima Ayyoubi Nezhad, Golnaz Tajeddin, Toktam Khatibi, Masoudreza Sohrabi

**Affiliations:** 1 School of Industrial and Systems Engineering, Tarbiat Modares University (TMU), Tehran, Iran; 2 Gastrointestinal and Liver Diseases Research Center, Iran University of Medical Sciences (IUMS), Tehran, Iran; University of Southern California, UNITED STATES OF AMERICA

## Abstract

Identifying anatomical landmarks in endoscopic video frames is essential for the early diagnosis of gastrointestinal diseases. However, this task remains challenging due to variability in visual characteristics across different regions and the limited availability of annotated data. In this study, we propose a novel self-supervised learning (SSL) framework that integrates three complementary pretext task, colorization, jigsaw puzzle solving, and patch prediction, to enhance feature learning from unlabeled endoscopic images. By leveraging these tasks, our model extracts rich, meaningful representations, improving the downstream classification of Z-line, esophageal, and antrum/pylorus regions. To further enhance feature extraction and model interpretability, we incorporate attention mechanisms, transformer-based architectures, and Grad-CAM visualization. The integration of attention layers and transformers strengthens the model’s ability to learn discriminative and generalizable features, while Grad-CAM improves explainability by highlighting critical decision-making regions. These enhancements make our approach more suitable for clinical deployment, ensuring both high accuracy and interpretability. We evaluate our proposed framework on a comprehensive dataset, demonstrating substantial improvements in classification accuracy, precision, recall, and F1-score compared to conventional models trained without SSL. Specifically, our combined model achieves a classification accuracy of 98%, with high precision and recall across all classes, as reflected in ROC curves and confusion matrices. These results underscore the effectiveness of pretext-task-based SSL, attention mechanism, and transformers for anatomical landmark identification in endoscopic video frames. Our work introduces a scalable and interpretable methodology for improving endoscopic image classification, reducing reliance on large annotated datasets while enhancing model performance in real-world clinical applications. Future research will explore validation on diverse datasets, real-time diagnostic integration, and scalability to further advance medical image analysis using SSL.

## 1. Introduction

The rapid advancement in machine learning (ML) and deep learning (DL) have significantly transformed computer vision, leading to breakthrough in medical image analysis. ML, a subset of artificial intelligence (AI), enables machines to recognize patterns and make data-driven decisions with minimal human intervention. Traditional ML techniques rely on manual feature extraction, which is time-consuming and error-prone. In contrast, DL models leverage hierarchical neural networks to automatically extract features and discern complex patterns, achieving superior performance in image classification, object detection, and segmentation [1].

In gastrointestinal (GI) endoscopy, accurate classification of abnormalities is essential for early diagnosis and treatment. However, conventional supervised deep learning models require large, labeled datasets, which are costly and labor-intensive to annotate. The scarcity of labeled medical images poses a significant challenge, particularly in resource-limited clinical setting. To address this, self-supervised learning (SSL) has emerged as a promising alternative, allowing models to learn meaningful representations from unlabeled data through pretest tasks.

Despite its success in general computer vision, SSL’s application in endoscopic classification remains limited. Existing studies primarily focus on single-task pretext learning, such as rotation prediction [2] or contrastive learning [3], and often rely solely on convolutional neural networks (CNNs). These approaches lack an effective multi-task strategy to maximize feature extraction and do not fully leverage attention mechanisms or transformer-based architectures, which have demonstrated superior performance in medical imaging. Moreover, many deep learning models function as “black boxes”, making it difficult for clinicians to interpret the decision-making process. Transparent AI models are crucial in medical applications, where understanding why a model makes a particular classification is essential for clinical trust and adoption.

To bridge these gaps, we propose a multi-task SSL framework for endoscopic image classification. Our approach integrates three complementary pretext tasks—colorization, patch prediction, and jigsaw puzzle solving—to enhance feature learning and classification performance. Additionally, we incorporate Grad-CAM (Gradient-weighted Class Activation Mapping) and SHAP (Shapley Additive Explanations) to improve interpretability, enabling clinicians to visualize the key regions that influence the model’s decisions.

**Key Contributions:**

- Multi-task SSL: We integrate colorization, patch prediction, and jigsaw puzzle solving to improve feature extraction in endoscopic images.
- Transformer-based feature extraction: Unlike conventional CNN-based approaches, we incorporate attention mechanisms to capture both local fine-grained details and global contextual dependencies.
- Robust model regularization: We employ dropout, weight decay, Synthetic Minority Over-sampling Technique (SMOTE), and early stopping to prevent overfitting and enhance generalization.
- Model interpretability with Grad-CAM and SHAP: We leverage Grad-CAM to generate heatmaps highlighting critical image regions and SHAP to quantify feature importance, ensuring transparent AI decision-making for clinical use.

To emphasize the novelty of our approach, Table 1 presents a comparative analysis of prior research on endoscopic image classification, focusing on methodologies, challenges addressed, and outcomes.

**Table 1:** Benchmarking endoscopic image classification techniques: A comparative analysis.

Study	Methodology	Challenges Addressed	Outcomes
[4]	Self-supervised patch-based matching in MCCE image stitching using a Transformer model	Limited shooting range, fragmented images, weak texture, close-up shooting, large angle rotation	Enhanced accuracy in addressing image parallax, improved NCM by up to 203%, improved SR by 55.8%
**[** **5** **]**	Domain-adaptive pre-training of foundation models with self-supervision on generic image data, followed by training on specific datasets	Variability of images, need for expert annotations, scarcity of large, high-quality labeled datasets	Achieved Macro AUC of 0.762 and balanced accuracy of 37.1% on the test set
**[** **6** **]**	Multi-modal multi-label Generalized Zero Shot Learning (GZSL) approach with feature disentanglement and multi-modal information	Predicting both seen and novel unseen disease classes in chest X-rays with multiple labels	Outperformed competing methods in experiments on NIH and CheXpert datasets, generating realistic multi-label disease samples of seen and unseen classes
**[** **7** **]**	Semi-supervised Strong-Teacher Consistency Network for few-shot multi-class cardiac MRI image segmentation	Limited labeled data for cardiac MRI, largely varying cardiac structures and anatomical features	Achieved 90.14% accuracy on MM-WHS and 78.45% accuracy on ACDC with 25% and 1% labeling rates, respectively
**Our study**	Multi-task SSL with attention layers, transformers, dropout, early stopping, SMOTE	Data scarcity, class imbalance, generalization, overfitting	Enhanced classification performance with reduced labeled data dependency

As shown in Table 1, existing approaches primarily focus on single-task self-supervised learning, domain adaptation, or semi-supervised techniques. These methods often require extensive labeled datasets or struggle with generalization to unseen data. In contrast, our proposed multi-task SSL leverages transformers, attention layers, and multiple pretext tasks to enhance feature extraction, reduce overfitting, and improve classification performance on endoscopic images.

This study presents an SSL framework that enhances endoscopic image classification by leveraging multiple pretext tasks. Unlike conventional supervised, zero-shot, and few-shot learning models, our approach incorporates attention layers, transformers, and regularization strategies to improve both accuracy and generalizability.

The subsequent sections provide an overview of related work, a detailed explanation of the methodology, experimental setup, results, and discussion on the effectiveness of the proposed framework.

## 2. Related works

ML, a subset of AI, has become a cornerstone of medical imaging, aiding in tasks such as image classification, feature extraction, and anomaly detection. Traditional ML models rely on hand-crafted features, requiring domain expertise to define relevant characteristics for classification. However, Deep Learning (DL) has revolutionized the field by leveraging multi-layered architectures that automatically extract hierarchical features directly from data. DL has demonstrated remarkable success across various imaging modalities, including X-ray [8], computed tomography (CT) [9], magnetic resonance imaging (MRI) [7], ultrasound [10], dermoscopy [11], endoscopic imaging [12], and multispectral or hyperspectral imaging [13–15].

Despite its success, DL models require large annotated datasets, a fundamental limitation in medical imaging where expert-annotated data is scarce. This constraint has fueled interest in SSL, which learns meaningful feature representations from unlabeled data. Unlike conventional supervised learning, SSL enables models to learn from inherent image structures, reducing dependency on manual annotations.

SSL methods aim to overcome annotation bottlenecks by deriving supervision from unstructured data. Early approaches, such as autoencoders and generative adversarial networks (GANs), focused on tasks like denoising, inpainting, and colorization [16,17]. These techniques allowed models to reconstruct missing parts of images, providing an unsupervised way to learn feature representations. However, such approaches often captured only low-level pixel dependencies rather than high-level semantic features essential for medical applications.

To address this, more advanced SSL techniques have emerged, such as contrastive learning [18] and context-based pretext tasks like jigsaw puzzles [19]. Contrastive learning maximizes the similarity between different views of the same image while pushing apart representations of different images, leading to more discriminative features. However, it requires large batch sizes and complex negative pair sampling strategies, which can limit practical implementation in medical settings. Jigsaw puzzles, on the other hand, force the model to learn spatial relationships within images, making it useful for structural learning but less effective in capturing fine-grained details.

Another category of SSL methods leverages temporal coherence in video data for representation learning. For example, a Siamese-triplet network with ranked loss functions was proposed to learn visual similarities from unlabeled video sequences [20]. However, these methods are primarily designed for natural videos and may not generalize well to static medical images, where temporal cues are absent.

In the specific domain of endoscopic image analysis, SSL has gained traction for learning representations from large-scale but unlabeled datasets. For example, contrastive loss-based approaches have been employed for helicobacter pylori detection [21], improving classification performance by learning discriminative features. Additionally, curriculum learning frameworks have been applied to classify HyperKvasir images [22], gradually increasing task complexity to enhance model robustness. However, these studies predominantly focus on a single SSL task, which limits the model’s ability to capture diverse anatomical variations in endoscopic images.

Another study introduced constrained contrastive distribution learning (CCD) for anomaly detection in colonoscopy images [16], demonstrating SSL’s effectiveness in rare event detection. Similarly, a Res-Unet based approach was applied for image restoration of gastrointestinal stromal tumors (GISTs) on multicenter endoscopic ultrasound (EUS) images [17], indicating SSL’s utility beyond classification tasks. Furthermore, rotation prediction was explored to assess SSL’s role in clinical diagnosis for wireless capsule endoscopy [23], but its effectiveness in multi-task learning remains underexplored.

A recent study proposed a two-step SSL framework for endoscopic video data [24], where embeddings were first extracted using pseudo-labels and triplet loss, then fine-tuned with limited labeled data. Although this method improved feature learning, it relied on video sequences, limiting its applicability to still endoscopic images. Similarly, a curriculum SSL framework based on Mixup and SimSiam was introduced for HyperKvasir image classification [22] leveraging augmentation strategies to enhance interpretability. However, these studies lack a systematic evaluation of multiple SSL tasks in combination, which is crucial for capturing both structural and textural variations in endoscopic images.

Building upon these advancements, our work introduces a novel multi-task SSL framework that integrates colorization, jigsaw puzzle solving, and patch prediction into a unified model. Unlike existing methods that focus on single-task SSL, our approach leverages the complementary strengths of multiple pretext tasks to learn richer feature representations. Colorization forces the model to understand color-based structural cues, jigsaw puzzle solving enhances spatial reasoning, and patch prediction improves texture understanding. Through extensive evaluation, we demonstrate that this combined approach outperforms individual pretext-task-based models and traditional supervised learning, offering a significant step forward in medical image analysis.

## 3. Materials and methods

In the following subsections, the main steps of the proposed method are explained. This includes the detailed description of the dataset used, the preprocessing steps applied to the data, the design and architecture of the proposed models, and the training and evaluation protocols. The pretext tasks utilized in the method, such as colorization, patch prediction, and jigsaw puzzle solving, are also discussed, highlighting their contribution to feature learning.

Additionally, the computational environment used to implement and validate the models is outlined to ensure reproducibility. The experiments were conducted using Google Colab, with the maximum available hardware resources, including 25.45 GB of RAM and 107.72 GB of disk space. The model training and evaluation leveraged GPU acceleration, utilizing NVIDIA K80, P100, P4, T4, and V100 GPUs provided by the platform. The entire implementation was carried out using Python, relying on libraries such as Scikit-learn, PyTorch, and OpenCV for data processing, model design, and evaluation.

### 3.1. Study design and overview

**Fig 1:**
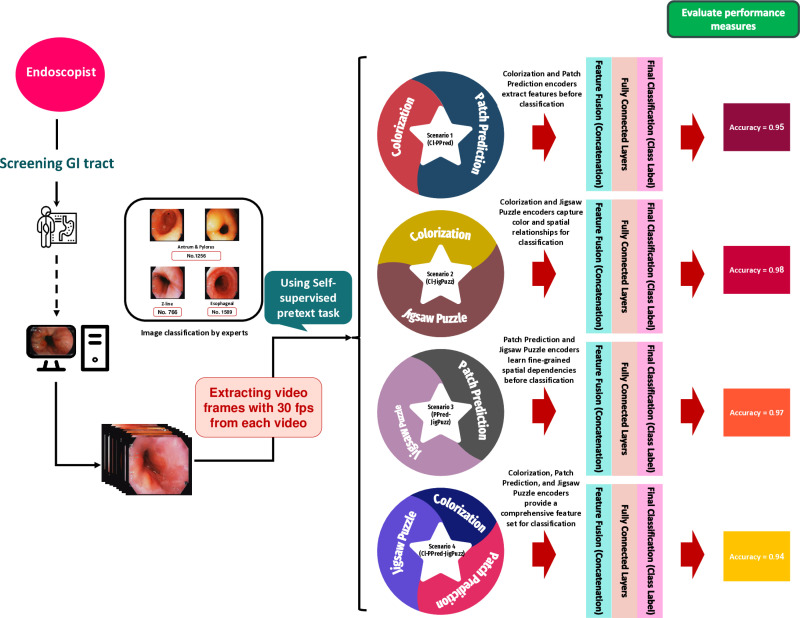
The main steps of the proposed scenarios for anatomical landmark detection from endoscopic video frames.

The aim of this study is to explore the effectiveness of SSL in classifying endoscopic images by leveraging three distinct pretext tasks: colorization, patch prediction, and jigsaw puzzles. These SSL tasks were employed to generate meaningful feature representations from unlabeled data, which were then utilized to enhance downstream classification performance. To evaluate their impact, four scenarios were designed, combining these pretext tasks in different configurations, as illustrated in Fig 1.

### 3.2. Ethics statement and dataset

This prospective study was conducted at the Endoscopy Department of Firoozgar Hospital, Tehran, Iran, between April 21, 2024 and June22, 2024. A total of 40 patients referred for endoscopy due to stomach pain were recruited. Before participation, all patients provided written informed consent after receiving a detailed explanations of the study’s purpose and procedures from attending physicians, in accordance with the Declaration of Helsinki. No minors were included in the study.

The study protocol was approved by the Ethics Committee of Tarbiat Modares University (Approval No. IR.MODARES.REC.1402.049, dated May 28, 2023). Endoscopic videos were collected as part of routine clinical procedures, anonymized before analysis, and securely stored to maintain confidentiality. Researchers had no access to identifying patient information at any stage of the study.

#### 3.2.1. Data description.

The dataset consists of endoscopic videos recorded at 30 frames per second (fps) from 40 patients. Due to variations in video length, the number of frames extracted per patient ranged from 3,600–54,000, yielding a total of 3,611 high-quality images. These images were manually reviewed and classified under except supervision into three anatomical categories: Z-line, esophageal, antrum/pylorus.

**Fig 2:**
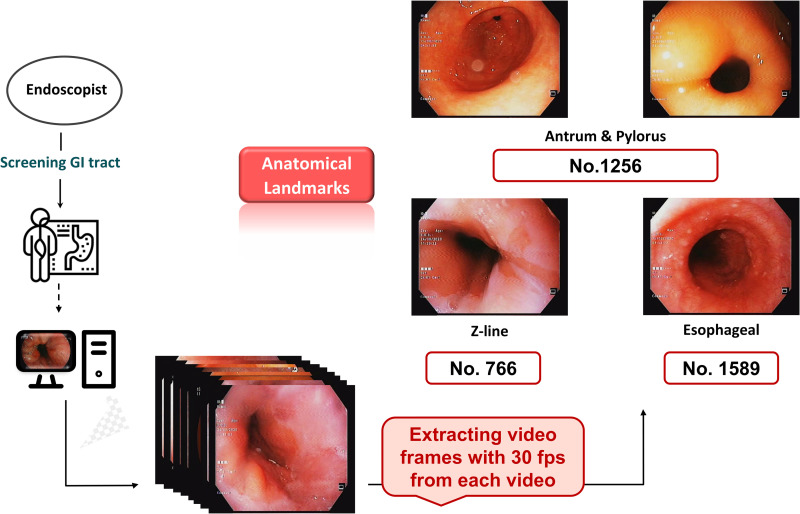
Data description of anatomical landmarks and tasks derived from endoscopic video frames.

Frames were extracted using the OpenCV Python library. Only frames containing clear anatomical landmarks were retained for classification. The resolution of each image was 576 × 768 pixels. The class distribution is presented in Fig 2, showing 1,256 images for the antrum/pylorus, 1,589 images for the esophageal, and 766 images for the Z-line category.

To ensure unbiased model evaluation and prevent data leakage, we implemented a patient-wise data split strategy during training, validation, and testing. This ensured that all frames from a single patient were exclusively assigned to one of these sets, preventing the model from memorizing patient-specific features and enabling a more generalizable assessment of its performance on unseen data.

#### 3.2.2. Data preparation.

All extracted images were resized to fixed dimension of 128 × 128 pixels to ensure uniform input size. The dataset was split into training (80%) and testing (20%) sets, with 2,888 images allocated for training and 723 images for testing.

**Handling class imbalance**

The dataset exhibited class imbalance, with a notably lower representation of the Z-line category compared to other classes. Such imbalance could lead to biased learning, where the model favors majority classes while underperforming on minority-class predictions.

To address this issue, we applied SMOTE to balance the training dataset by generating synthetic samples of the Z-line class. SMOTE creates new samples by interpolating between existing minority-class instances, increasing dataset diversity without simply duplicating existing images.

Importantly, SMOTE was applied exclusively to the training set to prevent information leakage into the test set, ensuring a fair evaluation of the model’s ability to classify unseen data accurately. After balancing, the training dataset achieved a more uniform class distribution, allowing the model to learn representative features across all anatomical categories.

To evaluate the impact of this balancing strategy, we re-executed all previous and newly designed evaluation scenarios. The results, presented in section 4. demonstrate improved classification performance for the Z-line class, with reduced misclassification rates and enhanced overall model robustness.

#### 3.2.3. Dataset limitations and future directions.

Although this dataset provides a diverse range of endoscopic images, we acknowledge certain limitations:

• All images were acquired from a single medical center using a specific endoscopic system, which may introduce biases related to imaging conditions and patient demographics.
• Anatomical variations across different patient populations could impact the generalization of the trained model.

To mitigate these challenges, we implemented extensive data augmentation techniques, including random rotations, flips, zooming, intensity adjustments, and contrast modifications, to simulate variations in imaging conditions and patient anatomy. Furthermore, SMOTE was used to address class imbalance, particularly improving representation for the Z-line category.

Despite these efforts, we recognize that incorporating data from multiple institutions and different endoscopic devices would further enhance model generalizability. However, similar sample sizes have been successfully utilized in previous medical imaging studies. For example, Byrne et al. demonstrated the feasibility of deep learning-based polyp detection using a dataset of 40 patients [18], highlighting that datasets of this scale can serve as proof-of-concept studies in medical image analysis.

This study serves as a proof-of-concept, validating the effectiveness of SSL for endoscopic image classification. Future work will focus on expanding the dataset and validating model performance across broader and more diverse real-world clinical settings.

### 3.3. SSL tasks (pretext tasks)

To enable robust feature learning from endoscopic images without the need for manual labels, we implemented three SSL pretext tasks: colorization, patch prediction, and jigsaw puzzle solving. The proposed framework incorporates different combinations of these tasks to evaluate their effectiveness in learning meaningful image representations.

The workflow of the proposed SSL approach is illustrated in Fig 1. The process begins with the acquisition of endoscopic video footage during GI tract screening, followed by expert classification of extracted frames into anatomical regions of interest (ROI), such as Z-line, esophageal, or antrum/pylorus. These classified images are then utilized for self-supervised training, where different pretext tasks are applied to encourage the model to learn useful representations.

We evaluate four distinct scenarios, each incorporating a unique combination of the three pretext tasks:

**Scenario 1 (CI-PPred):** Combines colorization and patch prediction.

**Scenario 2 (CI-JigPuzz):** Combines colorization and jigsaw puzzle solving.

**Scenario 3 (PPred-JigPuzz):** Combines patch Prediction and jigsaw puzzle solving.

**Scenario 4 (CI-PPred-JigPuzz):** Incorporates all three tasks.

Each scenario is assessed based on its ability to enhance feature extraction and improve downstream classification performance. The classification accuracy for each scenario is displayed on the right-hand side of Fig 1, with result ranging from 0.94 to 0.98, highlighting the effectiveness of these tasks in extracting meaningful features.

In the following sections, we provide a detailed mathematical formulation for each pretext task, along with the respective architectures used for training.

#### 3.3.1. Colorization task.

The goal of the image colorization task is to predict the color information of a grayscale image by learning meaningful representations of texture, edges, and object structures [25]. Given a grayscale input image, the model reconstructs the original color image by predicting the chrominance channels (a and b) from the luminance channel L. This forces the model to understand high-level semantic features, such as object boundaries and contextual cues.


$$I_{L}\in {\mathbb{R}}^{H\times W\times 1}$$ (1)

Let the input grayscale image be represented as:

where H and W denotes the height and width of the image, respectively. The corresponding ground truth color image consists of chrominance channels:


$$I_{ab}\in \;{\mathbb{R}}^{H\times W\times 2}\;$$ (2)

The network is trained to predict ${\hat{I}}_{ab}\in \;{\mathbb{R}}^{H\times W\times 2}$, representing the reconstructed color information.

To optimize the learning process, we use a pixel-wise mean squared error (MSE) loss function, ensuring minimal Euclidean distance between the predicted and actual color values:


$$L_{colorization}=\;\frac{1}{H\;\times W}\;\sum_{i=1}^{H}\sum_{j=1}^{W}\left({\left(I_{ab}(i,j)-\;{\hat{I}}_{ab}(i,j)\right)}^{2}\right)\;$$ (3)

where $I_{ab}(i,j)$ represents the ground truth chrominance values at pixel (i,j), and ${\hat{I}}_{ab}(i,j)$ is the predicted chrominance values.

This task enhances the model’s ability to understand textures, patterns, and object boundaries by leveraging the relationships between grayscale intensity and color. Unlike the jigsaw puzzle task (Section 3.3.3), which focuses on spatial arrangement learning, colorization primarily enhances feature extraction related to texture and contextual construct.

The model’s architecture and hyperparameters were optimized using a grid search approach, with details presented in Table 2.

**Table 2:** Architecture and parameters of autoencoder models for colorization task.

Layer	Output Shape	Parameters
Conv2d	(None, 16, 16, 64)	4160
ReLU	(None, 16, 16, 64)	0
Conv2d	(None, 8, 8, 128)	131200
ReLU	(None, 8, 8, 128)	0
Conv2d	(None, 4, 4, 256)	524544
ReLU	(None, 4, 4, 256)	0
Conv2d	(None, 2, 2, 512)	2097664
ConvTranspose2d	(None, 4, 4, 256)	2097664
ReLU	(None, 4, 4, 256)	0
ConvTranspose2d	(None, 8, 8, 128)	524544
ReLU	(None, 8, 8, 128)	0
ConvTranspose2d	(None, 16, 16, 64)	131200
ReLU	(None, 16, 16, 64)	0
ConvTranspose2d	(None, 32, 32, 3)	3075
Sigmoid	(None, 32, 32, 3)	0

#### 3.3.2. Patch prediction task.

The patch prediction task requires the model to infer the content of a missing patch given its surrounding patches, promoting contextual feature learning [26]. Let $I\in \;{\mathbb{R}}^{H\times W\times c}$ be an image divided into patches, where $P_{c}$ represents the missing central patch and $P_{1},\;P_{2},\;\ldots ,\;P_{k}$ are the surrounding context patches. The network learns to predict $P_{C}$ based on its context $I_{context}:$


$$L_{patch}=\;\frac{1}{p^{2}\times C}\;\sum_{i=1}^{p}\sum_{j=1}^{p}\sum_{c=1}^{c}{\left(P_{c}(i,j,c)-\;{\hat{P}}_{c}(i,j,c)\right)}^{2}\;$$ (4)

where ${\hat{P}}_{C}$ is the predicted patch. This reconstruction loss ensures the model accurately infers missing details from its surroundings.

Table 3 lists the architecture of the autoencoder model used for patch prediction, optimized via grid search.

**Table 3:** Architecture and parameters of autoencoder models for patch prediction task.

Layer	Output Shape	Parameters
Conv2d	(None, 16, 16, 64)	4160
ReLU	(None, 16, 16, 64)	0
Conv2d	(None, 8, 8, 128)	131200
ReLU	(None, 8, 8, 128)	0
Conv2d	(None, 4, 4, 256)	524544
ReLU	(None, 4, 4, 256)	0
Conv2d	(None, 2, 2, 512)	2097664
ConvTranspose2d	(None, 4, 4, 256)	2097664
ReLU	(None, 4, 4, 256)	0
ConvTranspose2d	(None, 8, 8, 128)	524544
ReLU	(None, 8, 8, 128)	0
ConvTranspose2d	(None, 16, 16, 64)	131200
ReLU	(None, 16, 16, 64)	0
ConvTranspose2d	(None, 32, 32, 3)	3075
Sigmoid	(None, 32, 32, 3)	0

#### 3.3.3. Jigsaw puzzle task.

The jigsaw puzzle task focuses on spatial understanding and global context reasoning by requiring the model to reassemble randomly shuffled image patches into their correct order [27]. This task forces the model to develop an understanding of object structures and spatial relationships, in contrast to the colorization task, which emphasizes color-based feature extraction.

An image $I\in \;{\mathbb{R}}^{H\times W\times C}$ is divided into n×n patches $P_{i}$. A random permutation π shuffles the patches, resulting in $I_{\pi }$. The model learns to predict the inverse permutation $\pi^{-1}$ to restore the correct spatial order:


$$L_{jigsaw}=\;-\;\sum_{i=1}^{n^{2}}y_{i}\log\left({\hat{y}}_{i}\right)\;$$ (5)

where $y_{i}$ is the true class label representing the correct permutation, and ${\hat{y}}_{i}$ is the predicted probability for that permutation.

Unlike colorization, which learns textural and fine-gained pixel-level details, the jigsaw puzzle task trains the model to recognize large-scale structures and object coherence. This complementary learning process ensures a robust feature representation, beneficial for downstream classification tasks.

The architecture and hyperparameters used for the task are presented in Table 4.

**Table 4:** Architecture and parameters of autoencoder models for jigsaw puzzle task.

Layer	Output Shape	Parameters
Conv2d	(None, 16, 16, 64)	4160
ReLU	(None, 16, 16, 64)	0
Conv2d	(None, 8, 8, 128)	131200
ReLU	(None, 8, 8, 128)	0
Conv2d	(None, 4, 4, 256)	524544
ReLU	(None, 4, 4, 256)	0
Conv2d	(None, 2, 2, 512)	2097664
ConvTranspose2d	(None, 4, 4, 256)	2097664
ReLU	(None, 4, 4, 256)	0
ConvTranspose2d	(None, 8, 8, 128)	524544
ReLU	(None, 8, 8, 128)	0
ConvTranspose2d	(None, 16, 16, 64)	131200
ReLU	(None, 16, 16, 64)	0
ConvTranspose2d	(None, 32, 32, 3)	3075
Sigmoid	(None, 32, 32, 3)	0

#### 3.3.4. Training and optimization.

Each pre-text tasks were trained for 20 epochs using the Adam optimizer, with a learning rate of 0.001 and a batch size of 32. The pretext trained models were later evaluated in downstream classification tasks to assess their effectiveness in learning useful representations from endoscopic images.

### 3.4. Proposed combined model

To leverage the strengths of multiple pretext tasks, we propose a novel combined model that integrates various autoencoders. This architecture aims to merge feature representations learned from colorization, patch prediction, and jigsaw puzzle tasks, enhancing overall robustness and accuracy in image classification.

By fusing complementary features extracted from different tasks, the model captures a richer understanding of image structures, leading to improved discriminative power. The integration strategy ensures that features learned from self-supervised tasks generalize well across different endoscopic image classes.

#### 3.4.1. Overview of Combined Model.

The proposed combined model is designed to synthesize features from three separate encoder networks, each trained on a distinct pretext task:

- **Colorization encoder –** Captures spatial and semantic dependencies by predicting color information from grayscale images.
- **Patch prediction encoder –** Learns to reconstruct missing patches, enhancing feature recognition of local textures and structural patterns.
- **Jigsaw puzzle encoder-** Train on shuffled image patches, reinforcing the model’s ability to understand global spatial relationships.

Each encoder independently extracts meaningful feature representations, which are aggregated and processed in the subsequent layers for classification tasks.

#### 3.4.2. Architecture details.

The combined model architecture consists of the following key components:

- **Encoders:** Feature extraction networks trained using individual pretext tasks.
- **Feature combination layer:** A fully connected layer that concatenates feature maps from all encoders.
- **Classification head:** A two-layer neural network that learns from the fused features to make the final prediction.

To construct the combined autoencoder, we first train each pretext task separately, then merge their learned representations. This strategy enables the model to retain specialized knowledge from each task while benefiting from a holistic understanding of the images.

Table 5 summarize the architecture variations for different integration scenarios.

**Table 5:** Architectural overview of combined autoencoder models for different pretext tasks.

Scenario	Architecture	Encoder	Decoder	Additional Components	Description of Layers and Parameters
**Scenario 1 (Cl-PPred)**	Combined Autoencoder with Colorization and Patch Prediction	Colorization Encoder, Patch Prediction Encoder	Patch Prediction Decoder	Linear Layer (Combined Features)	- **Encoder**:
				Classifier Head	Conv2d layers with ReLU activations
					Output channels: 64, 128, 256, 512
					Kernel size: 4
					Stride: 2
					Padding: 1
					- **Decoder**:
					ConvTranspose2d layers with ReLU activations
					Output channels: 3
					- **Additional**:
					Linear Layer to combine encodings (512 * 2 * 8 * 8–512)
					Classifier head (Linear, ReLU, Dropout)
**Scenario 2** **(Cl-JigPuzz)**	Combined Autoencoder with Colorization and Jigsaw	Colorization Encoder, Jigsaw Encoder	None (Only Classification Head)	Linear Layer (Combined Features)	- **Encoder**:
				Classifier Head	Conv2d layers with ReLU activations
					Output channels: 64, 128, 256, 512
					Kernel size: 4
					Stride: 2
					Padding: 1
					- **Classifier**:
					Linear layers (512 * 8 * 8–1024–3)
**Scenario 3** **(PPred-JigPuzz)**	Combined Autoencoder with Jigsaw and Patch Prediction	Jigsaw Encoder, Patch Prediction Encoder	None (Only Classification Head)	Linear Layer (Combined Features)	- **Encoder**:
				Classifier Head	Conv2d layers with ReLU activations
					Output channels: 64, 128, 256, 512
					Kernel size: 4
					Stride: 2
					Padding: 1
					- **Classifier**:
					Linear layers (512 * 8 * 8–1024–3)
**Scenario 4** **(CI-PPred-JigPuzz)**	Combined Autoencoder with Colorization, Patch Prediction, and Jigsaw	Colorization Encoder, Patch Prediction Encoder, Jigsaw Encoder	None (Only ClassificationHead)	Linear Layer (Combined Features)	- **Encoder**:
				Classifier Head	Conv2d layers with ReLU activations
					Output channels: 64, 128, 256, 512
					Kernel size: 4
					Stride: 2
					Padding: 1
					- **Classifier**:
					Linear layers (98304–512–3)
					Total features from all encoders concatenated

As seen in the Table 5, each architecture has been specifically designed to accommodate the unique requirements of its corresponding pretext task. The varying depth, layer configurations, and activation functions are intended to optimize performance for tasks. These design choices allow the combined model to learn rich, task-specific features that can later be leveraged for downstream classification tasks.

#### 3.4.3. Integration scenarios.

To evaluate different strategies for combining the pretext tasks, we explore four integration scenarios:

**Scenario 1 (CI-PPred)** Combines colorization and patch prediction encoders.

**Scenario 2 (CI-JigPuzz)** Merge colorization and jigsaw puzzle encoders.

**Scenario 3 (PPred-JigPuzz)** Integrates patch prediction and jigsaw puzzle encoders.

**Scenario 4 (CI-PPred-JigPuzz)** A fully integrated model incorporating all three pretext task encoders.

Each scenario follows a similar fusion process, but the variations in encoder combinations impact the feature diversity and classification accuracy. To further illustrate the integration approach, Fig 1. Presents a conceptual diagram of the combined model under each scenario. The visualization provides a structured representation of how different encoder combinations contribute to the final decision-making process.

Each scenario highlights a unique combination of self-supervised pretext tasks, where features extracted from individual encoders are concatenated and processed through fully connected layers for classification. The figure also depicts the role of feature fusion, demonstrating how varying levels of structural, spatial, and color-based features influence classification accuracy.

By examining each scenario’s accuracy and architectural setup, this visualization offers a clear understanding of the trade-offs and advantages of different encoder combinations in the proposed framework.

#### 3.4.4. Regularization and optimization strategies.

To prevent overfitting and improve generalization, the following regularization techniques were implemented:

- **Dropout layers**- Applied within the classification head to reduce over-reliance on specific neurons.
- **Early stopping**- Used to halt training once the validation loss plateaued, ensuring optimal model selection.
- **Data augmentation techniques**- Employed random rotations, flips, zooming, and intensity variations to improve model robustness.

These optimizations contributed to enhanced model stability reducing performance fluctuations.

### 3.5.. Self-supervised baseline comparison

To benchmark our proposed framework, we implemented SimCL, a state-of-the-art contrastive learning method, as a baseline SSL approach. SimCLR learns feature representations by maximizing agreement between augmented views of the same image while contrasting dissimilar instances.

**Fig 3:**
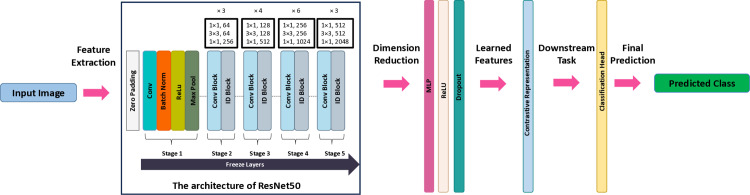
Architecture of the ResNet50-based self-supervised learning framework for anatomical landmark classification in endoscopic images.

Fig 3 illustrates the architecture of the ResNet50-based self-supervised learning (SSL) framework adapted for anatomical landmark classification in endoscopic images.

The figure consists of:

- **Backbone architecture**: ResNet-50 served as the encoder, with its final fully connected layer replaced by a **projection head** (two fully connected layers with ReLU activation and dropout).
- **Classification head**: A single fully connected layer mapped the learned 128-dimensional embeddings to the three anatomical classes: Z-line, esophageal, and antrum/pylorus.
- **Optimizer**: Adam (learning rate = 1×$10^{-4}$, weight decay = 1×$10^{-5}$,).
- **Loss function**: Label-smoothed cross-entropy to reduce overconfidence and improve generalization.
- **Scheduler**: ReduceLROnPlateau dynamically adjusted the learning rate based on validation loss.
- **Augmentation**: Random cropping, flipping, rotation, and contrast adjustments were applied to enhance robustness.

This setup ensured fair comparison with our multi-task framework while adhering to SimCLR’s standard practices for medical image analysis.

## 4. Results

The performance of the proposed models in identifying anatomical landmarks from endoscopic video frames was evaluated using multiple scenarios. Each scenario combines different pretext tasks, including colorization (Cl), patch prediction (PPred), and jigsaw puzzle (JigPuzz). The metrics used to assess the performance include accuracy, weighted average precision, weighted average recall, weighted average F1-score, macro average precision, macro average recall, and macro average F1-score.

### 4.1. Performance evaluation of pretext task combination

Table 6 summarizes the performance of each scenario across these metrics.

**Table 6:** The performance measures of the proposed scenarios for anatomical landmark identification from endoscopic video frames.

Performance metrics	Scenario 1 (Cl-PPred)	Scenario 2 (Cl-JigPuzz)	Scenario 3 (PPred-JigPuzz)	Scenario 4 (Cl-PPred-JigPuzz)
Accuracy	95.00	**98.00**	97.00	94.00
Weighted avg-Precision	96.00	**98.00**	97.00	94.00
Weighted avg-Recall	96.00	**98.00**	97.00	94.00
Weighted avg-F1-Score	96.00	**98.00**	97.00	94.00
Macro avg-Precision	96.00	**98.00**	97.00	94.00
Macro avg-Recall	96.00	**98.00**	97.00	94.00
Macro avg-F1-Score	96.00	**98.00**	97.00	94.00

To further improve model generalization and mitigate potential overfitting, we incorporated regularization techniques, dropout layers, SMOTE, data augmentation, and early stopping into the training process. These enhancements aimed to increase robustness and ensure stable performance across different scenarios.

After implementing these strategies, we re-evaluated the proposed scenarios and observed notable improvements in performance metrics. Table 6 presents the revised results, demonstrating the effectiveness of these techniques in enhancing classification accuracy and stability.

Overall, these refinements resulted in improved classification measures particularly in Scenario 2 (Cl-JigPuzz). The results confirm that integrating these optimization techniques enhances the reliability of anatomical landmark identification from endoscopic video frames.

**Fig 4:**
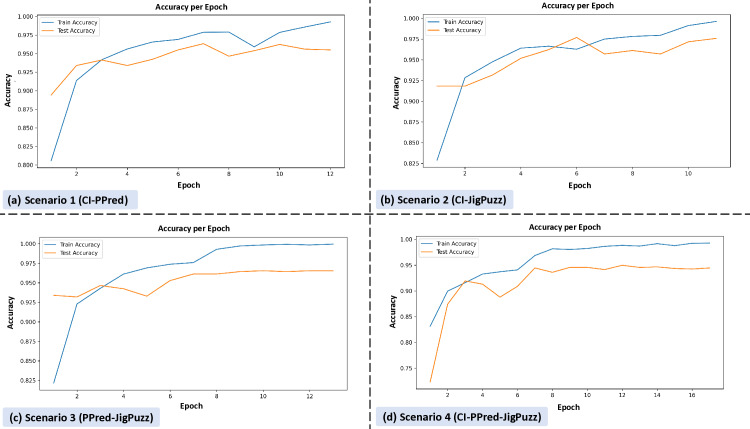
Training accuracy per epoch for different pretext task combinations. Subplot (a) shows Scenario 1 (CI-PPred), with training accuracy increasing steadily to near 1.0 and test accuracy showing slight fluctuations. Subplot (b) depicts Scenario 2 (CI-JigPuzz), with both training and test accuracy showing a consistent upward trend, with test accuracy stabilizing around 0.95. Subplot (c) illustrates Scenario 3 (PPred-JigPuzz), with training accuracy rising quickly and test accuracy showing steady improvement. Subplot (d) represents Scenario 4 (CI-PPred-JigPuzz), with training accuracy reaching near 1.0 and test accuracy showing gradual improvement, stabilizing around 0.95.

Fig 4 comprises four subplots, each representing the accuracy per epoch for different scenarios.

**Fig 5:**
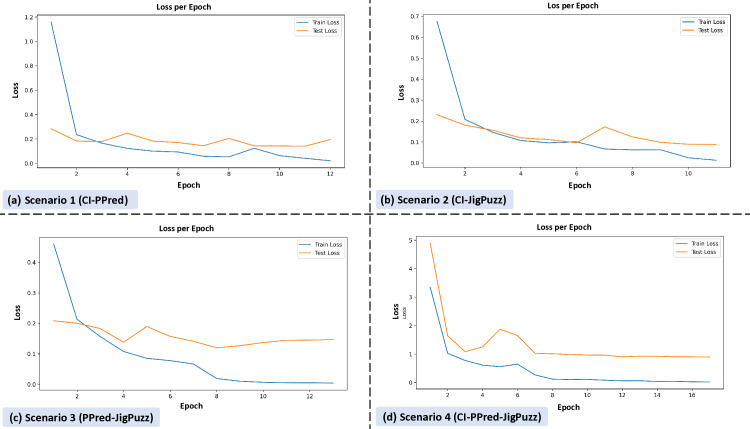
Training and validation loss per epoch for different pretext task combinations. Subplot (a) shows Scenario 1, where training loss decreases rapidly and stabilizes, with test loss following a similar trend. Subplot (b) depicts Scenario 2, where both training and test loss decrease rapidly initially and then gradually, with training loss generally lower than test loss. Subplot (c) illustrates Scenario 3, with training loss decreasing quickly and stabilizing, while test loss follows a similar trend but remains slightly higher. Subplot (d) represents Scenario 4, where training loss decreases rapidly and stabilizes, with test loss following a similar pattern but with higher initial values.

Fig 5 consists of four subplots, each representing the loss per epoch for different scenarios.

**Fig 6:**
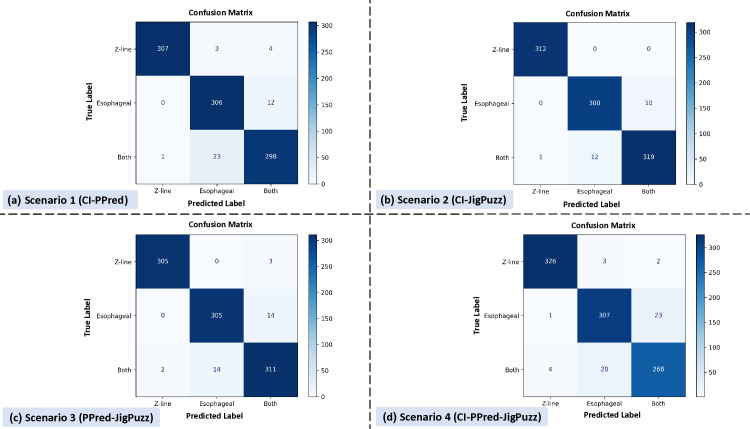
Confusion matrices for each pretext task combination. Each matrix is color-coded with a gradient from light blue to dark blue, indicating frequency. In matrix (a), Scenario 1 shows high accuracy for true Z-line and esophageal categories. Matrix (b), Scenario 2 exhibits the highest accuracy among scenarios. Matrix (c), Scenario 3 shows high accuracy but with minor misclassifications. Matrix (d), Scenario 4 demonstrates lower accuracy with more misclassifications compared to other scenarios.

Fig 6 shows four confusion matrices labeled (a), (b), (c), and (d), each comparing predicted labels against true labels for three categories: Z-line, esophageal, and Both (antrum/pylorus).

**Fig 7:**
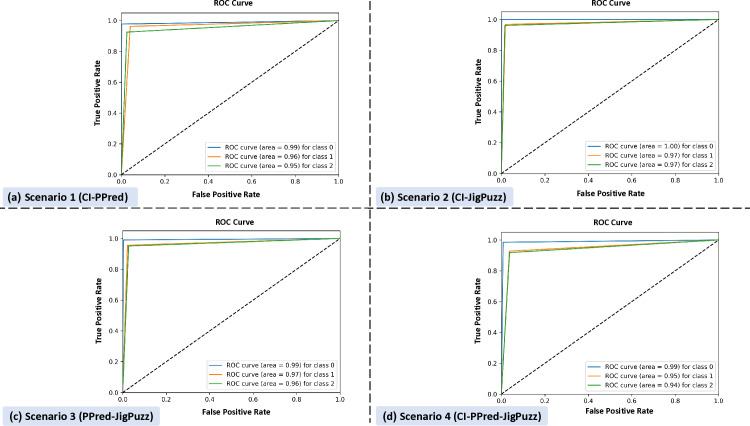
ROC curves for each pretext task combination, illustrating model performance across classes. Subplot (a), Scenario 1 indicates high performance, with areas of 0.99, 0.96, and 0.95. Subplot (b), Scenario 2 exhibits the highest accuracy, with areas of 1.00, 0.97, and 0.97. Subplot (c), Scenario 3 shows high performance, with areas of 0.99, 0.97, and 0.96. Subplot (d) indicates lower performance, with areas of 0.99, 0.95, and 0.94.

Fig 7 shows four ROC curves labeled (a), (b), (c), and (d), each plotting True Positive Rate (TPR) against False Positive Rate for three categories: class 0 (Z-line), class 1(esophageal), and class 2(antrum/pylorus).

### 4.2. Ablation study on individual pretext tasks

To assess the contribution of each pretext task independently, we conducted a detailed ablation study by designing three experimental scenarios:

- **Scenario 1 (Colorization-only):** The model was trained using only the colorization pretext task.
- **Scenario 2 (Jigsaw puzzle-only):** The model was trained using only the jigsaw puzzle pretext task.
- **Scenario 3 (Patch prediction-only):** The model was trained using only the patch prediction pretext task.

For each scenario, the classification performance was evaluated using accuracy, weighted and macro-averaged precision, recall, and F1-score. Table 7 presents the results of these experiments.

**Table 7:** The performance measures of each pretext task in isolation.

Performance metrics	Scenario 1 (Cl)	Scenario 2 (JigPuzz)	Scenario 3 (PPred)
Accuracy	95.00	94.00	95.00
Weighted avg-Precision	95.00	94.00	95.00
Weighted avg-Recall	95.00	94.00	95.00
Weighted avg-F1-Score	95.00	94.00	95.00
Macro avg-Precision	95.00	94.00	95.00
Macro avg-Recall	95.00	94.00	95.00
Macro avg-F1-Score	95.00	94.00	95.00

The results, as presented in Table 7, indicate that the colorization and patch prediction tasks achieved the highest performance, with an accuracy of 95%, while the jigsaw puzzle task performed slightly lower at 94%. This suggests that colorization and patch prediction contribute more to learning robust feature representations. However, the relatively small performance gap implies that all three pretext tasks contribute effectively to the overall model performance.

**Fig 8:**
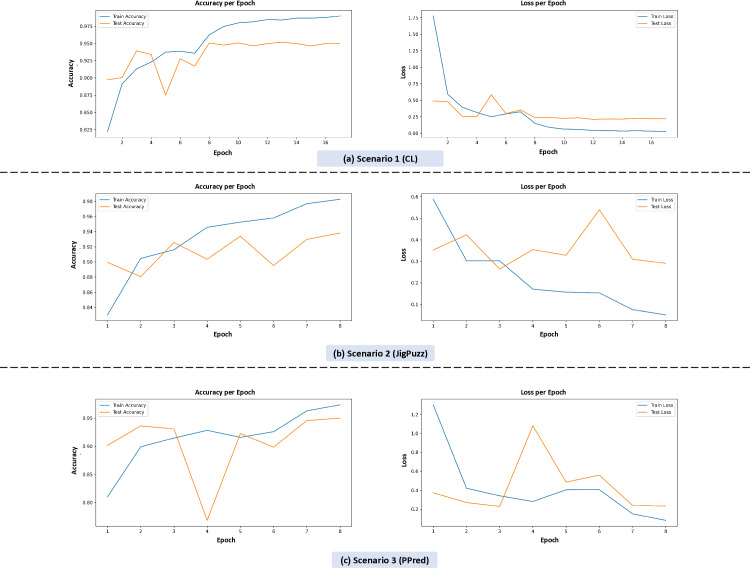
Training accuracy and loss per epoch for individual pretext tasks (colorization, jigsaw puzzle, patch prediction).

Fig 8 illustrates the accuracy and loss per epoch for each scenario, providing insights into the training dynamics and convergence behavior.

In Scenario 1 (Colorization-only), the model achieved a high initial accuracy, which stabilized around 0.975 after a few epochs. The loss per epoch decreased steadily, indicating effective learning and convergence. Similarly, in Scenario 3 (Patch Prediction-only), the model demonstrated consistent accuracy, reaching up to 0.95, with loss decreasing smoothly over epochs. This suggests that both colorization and patch prediction tasks facilitate robust feature learning, likely due to their ability to capture semantic and structural information in endoscopic images.

In contrast, Scenario 2 (Jigsaw Puzzle-only) showed a slightly lower accuracy, peaking at 0.94, with more variability in the loss per epoch. This indicates that while the jigsaw puzzle task contributes to feature learning, it may require more epochs to stabilize compared to the other tasks.

By isolating the impact of each pretext task, this ablation study confirms that each task plays a role in feature learning, with colorization and patch prediction exhibiting slightly stronger contributions. These findings support our choice of combining multiple pretext tasks to enhance classification performance.

**Fig 9:**
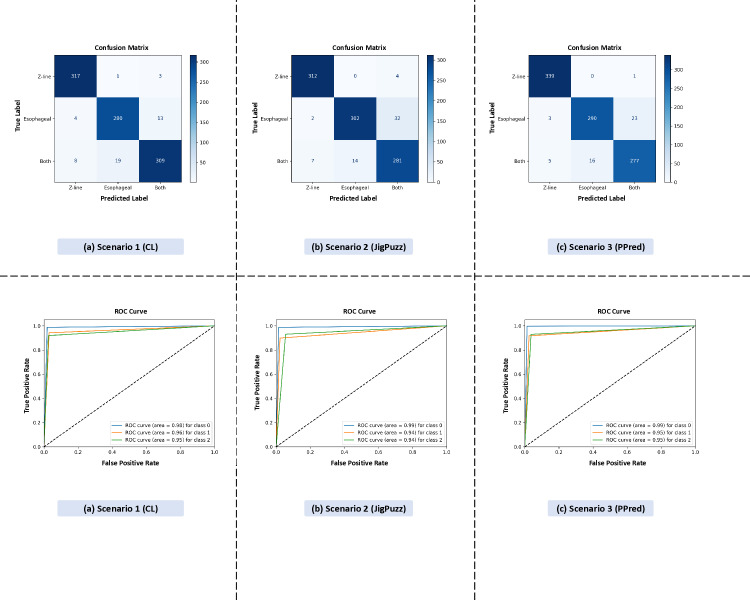
Comparison of classification performance for individual pretext tasks using confusion matrices and ROC curves.

Fig 9 consists of six subplots. The top row displays confusion matrices, while the bottom row shows ROC curves for three scenarios: Scenario 1 (CL), Scenario 2 (JigPuzz), and Scenario 3 (PPred).

These plots support the findings in Table 7, confirming the classification performance of each pretext task. The colorization and patch prediction tasks show slightly higher performance compared to the jigsaw puzzle task, aligning with the reported accuracy and other performance metrics. This confirms that colorization and patch prediction contribute more to learning robust feature representations, while all three pretext tasks collectively enhance overall model performance.

#### 4.2.1. Computational efficiency.

To evaluate the computational efficiency of each pretext task, we measured the processing time required for both training and inference. Table 8 presents the time taken for the forward and backward pass during training (20 epochs) as well as the forward pass during inference for each scenario.

**Table 8:** Processing time for each pretext task.

Scenarios	Processing steps	Processing time (Sec.)
Scenario 1 (Cl)	Forward + Backward Pass (20 Epochs)	156
Scenario 2 (JigPuzz)	Forward + Backward Pass (20 Epochs)	156
Scenario 3 (PPred)	Forward + Backward Pass (20 Epochs)	156
Scenario 1 (Cl)	Forward Pass	0.875
Scenario 2 (JigPuzz)	Forward Pass	0.875
Scenario 3 (PPred)	Forward Pass	0.875

The results in Table 8 indicate that training each pretext task separately requires 156 seconds for 20 epochs, while the inference time per sample remains low at 0.875 seconds. These results highlight the computational feasibility of individual pretext tasks, demonstrating that the training time remains reasonable even in resource-constrained environments. While the pretraining phase introduces additional computation compared to traditional supervised learning, the relatively short processing time suggests that these tasks can be effectively integrated into self-supervised learning frameworks without significant resource limitations. Furthermore, since pretext tasks are trained offline, their computational demands do not impact real-time inference. By leveraging GPU acceleration, we ensure that the additional computational overhead remains manageable. However, in resource-constrained environments such as portable endoscopic systems, strategies such as knowledge distillation, model pruning, and hardware optimization can be explored to further reduce computational costs while preserving model performance. These findings confirm that while integrating multiple pretext tasks increases computational complexity during training, the impact on inference speed is minimal, making the proposed approach suitable for practical applications in medical imaging.

### 4.3. Enhancing interpretability with attention, transformers, and Grad-CAM

To improve the interpretability of the proposed SSL framework for clinical deployment, we explored attention mechanisms, transformer-based architectures, and visualization techniques. These approaches aim to enhance feature extraction and provide better insights into the model’s decision-making process.

#### 4.3.1. Attention mechanism integration.

The attention mechanism was incorporated into the model to highlight critical spatial features in endoscopic images, enabling more precise localization of relevant anatomical structures. By weighting important regions, the model can focus on clinically significant areas, potentially improving diagnostic reliability. The results in Table 9 show that the attention-based model achieves comparable performance to the baseline, with an accuracy of 97%, while offering improved interpretability.

**Table 9:** The performance comparison of attention-based and transformer-based models.

Performance metrics	(Cl-JigPuzz) + Attention	(Cl-JigPuzz) + Transformer
Accuracy	97.00	97.00
Weighted avg-Precision	97.00	97.00
Weighted avg-Recall	97.00	97.00
Weighted avg-F1-Score	97.00	97.00
Macro avg-Precision	97.00	97.00
Macro avg-Recall	97.00	97.00
Macro avg-F1-Score	97.00	97.00

#### 4.3.2. Vision transformer (ViT) for feature extraction.

Inspired by recent advances in vision-based deep learning, we also integrated a transformer-based architecture (Vision Transformer, ViT) into the framework. The ViT model effectively captures long-range dependencies between image patches, facilitating better contextual understanding of anatomical landmarks.

#### 4.3.3. Grad-CAM for model decision visualization.

To further enhance the interpretability of the proposed framework, we employed Grad-CAM to visualize the key regions influencing the model’s predictions. Grad-CAM generates heatmaps that highlight the most influential areas in an image, offering a layer of explainability crucial for clinical applications [28].

**Fig 10:**
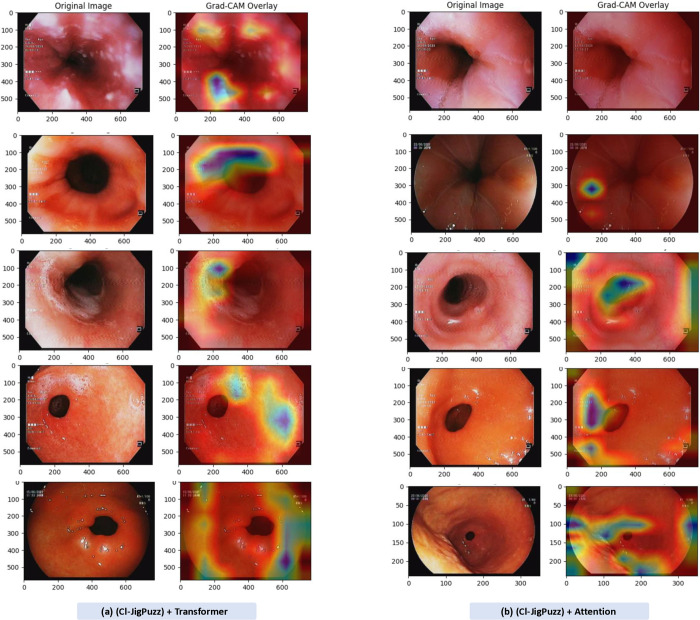
Grad-CAM visualization of model predictions highlighting clinically relevant features in endoscopic images.

Grad-CAM was applied to the final convolutional layers of our model, providing insight into whether the model attends to relevant anatomical structures or potential artifacts. Fig 10 illustrates the Grad-CAM visualizations, where heatmaps are overlaid on endoscopic images for different classes.

The visualizations confirm that the model primarily focuses on clinically relevant regions, such as mucosal patterns, lesion boundaries, and the Z-line, reinforcing its potential for clinical use.

#### 4.3.4. Comparison and clinical implications.

Despite achieving similar quantitative results (Table 9), the key advantage of these architectures lies in their interpretability. The attention heatmaps and Grad-CAM visualizations provide a visual representation of the model’s focus areas, offering potential clinical insights. Additionally, transformer-based models excel in learning hierarchical feature representations, which could aid in distinguishing subtle variations in endoscopic images.

The inclusion of Grad-CAM-based visualization techniques further enhances transparency, allowing clinicians to verify model predictions and ensure trustworthiness. These enhancements align with the goal of making AI-assisted diagnosis more explainable and reliable for clinical applications.

We evaluated the effect of these interpretability-driven enhancements on classification performance. As shown in Table 9, both modifications resulted in an accuracy of 97%, demonstrating that these enhancements maintain high classification performance while improving model interpretability. The inclusion of an attention layer and transformer block facilitates a more explainable decision-making process, essential for clinical applications.

**Fig 11:**
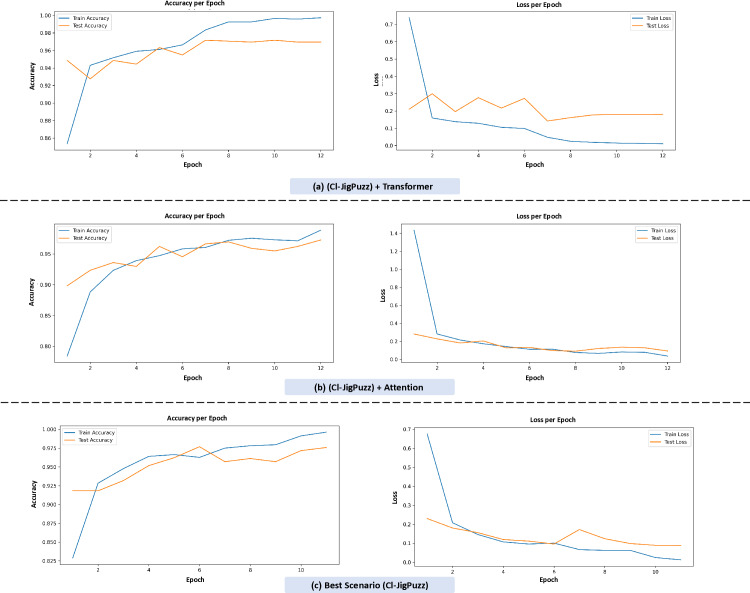
Training dynamics of multi-task self-supervised models: comparative analysis of architectural enhancements (Transformer vs. Attention) for endoscopic image classification.

Fig 11 illustrates the training dynamics of combined pretext tasks with different architectural mechanisms, evaluated through accuracy and loss trends across epochs.

Three configurations are compared:

- **(a) (CI-JigPuzz) + Transformer**: The model achieves near-perfect initial accuracy (1.00), maintaining high performance (≥0.86) as training progresses. The loss decreases smoothly from 0.7 to 0.0, indicating stable convergence.
- **(b) (CI-JigPuzz) + Attention**: Accuracy starts at 0.95 but declines to 0.80, accompanied by a more volatile loss curve (1.4 to 0.0), suggesting slower convergence and potential instability compared to the Transformer variant.
- **(c) Best scenario (CI-JigPuzz)**: This configuration demonstrates robust performance, with accuracy remaining high (1.000 to 0.875) and loss decreasing steadily (0.7 to 0.0), reflecting effective learning and generalization.

The results highlight that integrating the Colorization (CI) and Jigsaw Puzzle (JigPuzz) pretext tasks with a Transformer mechanism (a) yields strong stability, while the standalone combination (c) achieves the best balance of high accuracy and low loss. The comparative analysis underscores the importance of architectural choices in optimizing multi-task pretext learning, with the Transformer-enhanced configuration and the combined CI-JigPuzz setup showing superior efficacy for endoscopic image classification. These findings further validate the benefits of combining pretext tasks and advanced mechanisms to enhance feature representation learning.

**Fig 12:**
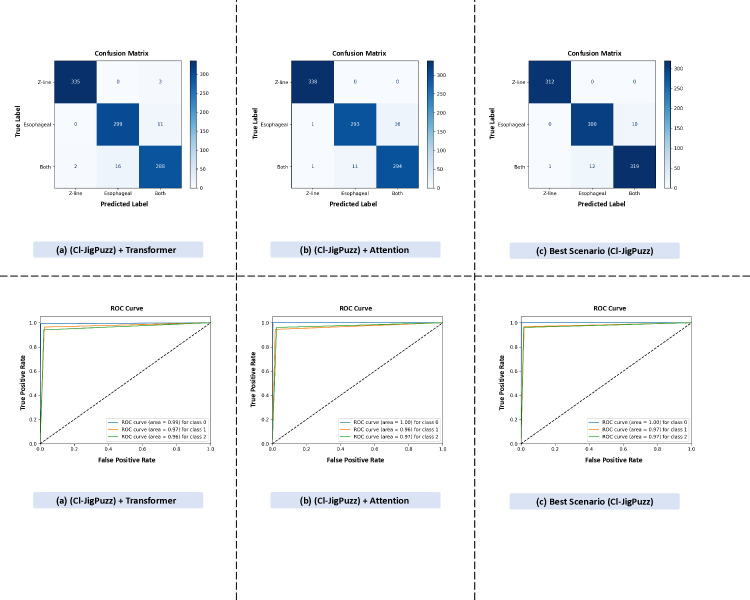
Comparison of classification performance across three models using confusion matrices and ROC curves. The figure illustrates accuracy and loss trends across training epochs for three model variations: (a) (CI-JigPuzz) + Transformer – The model demonstrates rapid convergence, achieving near-perfect initial accuracy (1.00) and maintaining high performance (≥0.86) throughout training. The loss curve steadily decreases from 0.7 to 0.0, reflecting stable optimization. (b) (CI-JigPuzz) + Attention – Initial accuracy starts at 0.95 but declines to 0.80 over epochs. The loss curve exhibits more volatility (1.4 to 0.0), indicating a slower convergence rate compared to the Transformer-based model. (c) Best Scenario (CI-JigPuzz) – This configuration achieves optimal balance, with accuracy consistently high (1.000 to 0.875) and loss decreasing smoothly (0.7 to 0.0), showcasing effective learning and generalization.

Fig 12 is training dynamics of combined pretext tasks under different architectural configurations.

These results highlight the impact of architectural choices in multi-task pretext learning. While the Transformer-based approach ensures stability, the combined CI-JigPuzz model achieves the best trade-off between accuracy and loss minimization. This further validates the significance of integrating multiple pretext tasks with advanced mechanisms for robust endoscopic image classification.

### 4.4. Comparison with SSL baseline

**Fig 13:**
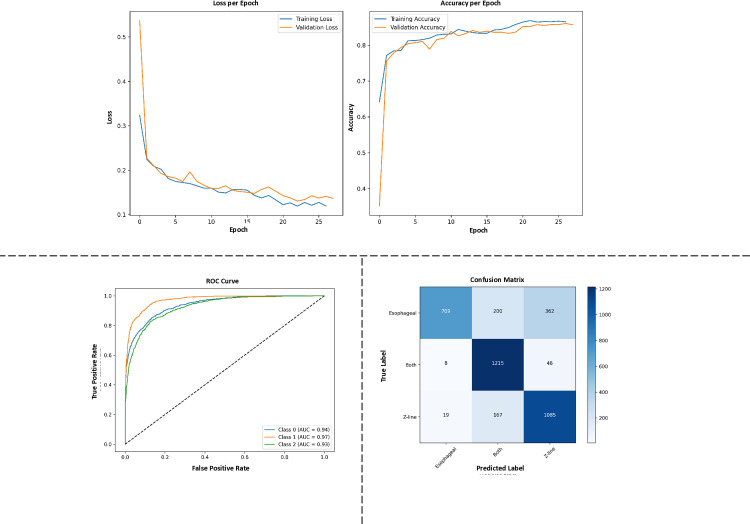
SimCLR baseline performance in endoscopic image classification.

To assess the effectiveness of our multi-task self-supervised framework, we compared it against a SimCLR-based model trained under identical conditions. Fig 13 illustrates the training dynamics, generalization, and discriminative performance of SimCLR, while Table 10 quantitatively contrasts its macro-averaged metrics against our framework.

**Table 10:** Performance comparison of the proposed multi-task self-supervised model and SimCLR (Macro-Averaged Metrics).

Model	Accuracy	Precision	Recall	F1-Score
Ours (Cl-JigPuzz)	98.00	98.00	98.00	98.00
SimCLR (Baseline SSL)	88.00	88.00	88.00	88.00

The loss curves (Training and Validation Loss) in Fig 13 show that SimCLR achieves near-zero validation loss by epoch 25, suggesting optimization convergence. However, the accuracy curves reveal a plateau at 88% validation accuracy, consistent with Table 10. This gap between low loss and suboptimal accuracy highlights SimCLR’s limitation in translating learned features into high classification performance, likely due to its reliance on contrastive learning alone.

The confusion matrix (Time Label vs. Predicted Label) exposes SimCLR’s challenges with minority classes. Misclassifications in these categories underscore its inability to learn fine-grained anatomical features critical for medical imaging, a weakness addressed by our multi-task framework through explicit pretext tasks.

The ROC curve for SimCLR reports a TPR with Mutual Information Coefficients (MIC) up to 0.93 at higher gains, indicating reasonable discriminatory power. However, these metrics do not fully compensate for its lower overall accuracy (88%), as anatomical variations in endoscopic images require more nuanced feature learning than contrastive methods provide.

While SimCLR’s training and validation loss curves align closely, its validation accuracy (88%) lags significantly behind our framework’s 98% (Table 10). This suggests that SimCLR learns generic features insufficient for robust medical image classification, despite stable optimization.

Fig 13 underscores SimCLR’s limitations in endoscopic image analysis. While it achieves decent MIC scores and loss convergence, its inability to classify minority classes (confusion matrix) and lower overall accuracy (vs. Table 10) reflect the shortcomings of contrastive learning in isolation. Our multi-task framework, by integrating pretext tasks like colorization (CI) and jigsaw puzzles (JigPuzz), explicitly addresses these gaps, enabling superior anatomical feature learning and generalization.

Although SimCLR serves as a strong self-supervised baseline, its reliance on contrastive learning alone may be less effective for medical image classification, where learning fine-grained anatomical features is crucial.

### 4.5. Model explainability

To enhance the interpretability of our proposed model and provide clinicians with insights into its decision-making process, we implemented SHAP in our best-performing scenario, which combines jigsaw puzzle and colorization pretext tasks. This combination demonstrated superior classification performance, and applying SHAP to this scenario allows us to understand the critical features that influence the model’s predictions [29].

**Fig 14:**
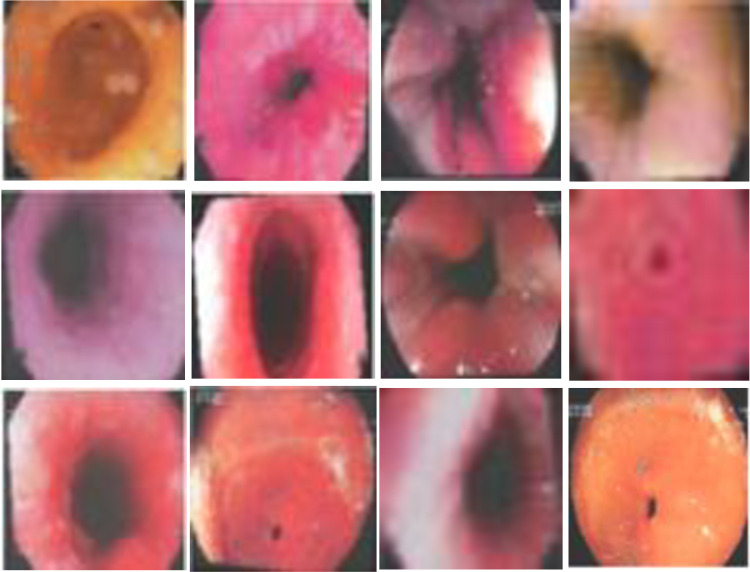
SHAP heatmaps.

We generated SHAP heatmaps for different classes, highlighting the most influential regions that contributed to the classification decision. The SHAP values were overlaid onto the original images, with a color gradient representing pixel importance, where higher-intensity areas indicate greater significance in the decision-making process. Fig 14 presents examples of their corresponding SHAP heatmaps, illustrating how the model attends to clinically relevant regions.

The results show that the model predominantly focuses on meaningful anatomical structures, such as mucosal patterns and pathological features, validating the effectiveness of the jigsaw-colorization combination. This explainability approach enhances model transparency, which is crucial for clinical adoption and trust in AI-based decision support systems.

## 5. Conclusion and discussion

This study presents a novel SSL framework utilizing multiple pretext tasks to enhance feature learning for endoscopic image classification. By integrating jigsaw puzzle solving, patch prediction, and colorization, our approach enables the model to extract meaningful representations from unlabeled data, addressing the challenge of limited annotated medical datasets. Our results demonstrate that pretext-task-based SSL offers distinct advantages over contrastive learning methods like SimCLR. While contrastive learning is effective in general representation learning, its reliance on contrastive loss is less suited for small medical datasets, where precise feature extraction from localized structures is critical. In contrast, our domain-specific pretext tasks guide the model towards clinically relevant feature representations, improving generalization across different anatomical landmarks.

The implications of our findings are significant for clinical practice, particularly in the automated diagnosis of esophageal diseases. By achieving high classification accuracy and reducing misclassification rates, our models could assist clinicians in identifying pathological regions with greater precision. Moreover, our approach reduces dependency on large annotated datasets, which is a major challenge in medical imaging research. The potential for deployment in resource-constrained settings, such as portable endoscopic systems, further enhances its practical value. Once trained, the model requires minimal computational resources for inference, making real-time applications feasible.

To further enhance the interpretability of our SSL framework, we integrated Grad-CAM visualization to highlight the regions that influenced the model’s predictions. The Grad-CAM heatmaps provide insightful visual representations of the anatomically significant areas that the model focuses on, helping clinicians understand the decision-making process. This interpretability is crucial for clinical deployment, as it builds trust in AI-assisted diagnostics and ensures that the model’s predictions align with expert medical knowledge. Our results demonstrate that attention-based methods, including transformers and Grad-CAM visualizations, improve transparency while maintaining high classification performance.

Despite these advantages, some challenges remain. The limited size and potential imbalance in the dataset pose difficulties for model performance. Expanding the dataset or applying advanced data augmentation techniques could mitigate these issues and further improve generalization. Additionally, the integration of multiple pretext tasks, while beneficial for feature learning, increases computational complexity during the pretraining phase. However, since our framework is designed for offline training, this does not impact real-time inference. Pretrained models can be periodically updated as new data becomes available, ensuring adaptability without the need for continuous high-resource computations.

To build upon the contributions of this study, future research should focus on the following areas:

1. **Dataset expansion and diversity** – While our method has demonstrated strong performance, validating it on larger and more diverse datasets, including multi-institutional and multi-modal endoscopic images, is crucial to ensure robustness across different patient populations and imaging conditions.
2. **Benchmarking against other SSL methods** – Although our study highlights the advantages of pretext-task-based learning, future work should include comparisons with additional SSL approaches, such as MoCo and SwAV, to comprehensively evaluate the effectiveness of different frameworks in medical imaging.
3. **Ablation studies for task contribution analysis** – Conducting detailed ablation studies will provide insights into the impact of each pretext task, allowing for a more refined and optimized SSL pipeline tailored to medical imaging applications.
4. **Optimization for real-time clinical use** – Reducing inference time and computational complexity is essential for practical deployment. Exploring techniques such as knowledge distillation and model quantization will allow us to compress the model while maintaining high accuracy, making it suitable for integration into real-time diagnostic workflows and edge computing environments.
5. **Clinical validation and prospective studies** – To ensure real-world applicability, our model should undergo prospective validation studies in collaboration with gastroenterologists. Evaluating its performance in real clinical settings will be essential for assessing its reliability, interpretability, and impact on diagnostic decision-making.

By addressing these future directions, our study provides a foundation for more robust and clinically applicable AI-driven diagnostic tools in gastroenterology. The findings underscore the potential of SSL in medical imaging and pave the way for further advancements in automated disease detection and classification. The integration of Grad-CAM further enhances model interpretability, ensuring that AI-assisted diagnostics are not only accurate but also explainable and trustworthy for clinical deployment.
